# An Exploratory Study of the Differences in Attitudes and Motives Regarding COVID-19 Plasma Donation

**DOI:** 10.15388/Amed.2022.29.1.14

**Published:** 2022-07-26

**Authors:** Ashish Maheshwari, Mohit Varshney, Meenu Bajpai, Neeraj Raizada, Tarika Sharma

**Affiliations:** Department of Transfusion Medicine, Institute of Liver and Biliary Sciences, New Delhi; Department of Psychiatry, Institute of Liver and Biliary Sciences, New Delhi; Department of Transfusion Medicine, Institute of Liver and Biliary Sciences, New Delhi; Department of Clinical Research and Epidemiology, Institute of Liver and Biliary Sciences, New Delhi; College of Nursing, Institute of Liver and Biliary Sciences, New Delhi

**Keywords:** plasma donors, plasma donation, attitudes, motives

## Abstract

**Background:**

Understanding the attitude and motives and differences between voluntary and replacement blood donation is the key to the sustainable availability of this precious resource. This study aimed to assess the attitude and motives for convalescent plasma (CP) donation in the recovered COVID-19 plasma donors and further understand the differences between voluntary and replacement donation.

**Materials and Methods:**

This prospective cross-sectional survey-based study was conducted among500 COVID-19 recovered blood donors who visited for CP donation at a tertiary care super-speciality centre in northern India. Data were collected using a structured questionnaire based on donor attitude, motives, and belief, which was validated by the experts of Psychiatry, Transfusion Medicine, and Epidemiology and was administered by the online medium.

**Results:**

The study’s findings depicted that voluntary plasma donors were previously regular blood donors (36.8%) compared to replacement plasma donors (26.4%). Almost all voluntary donors (99.5%) showed altruistic reasons to donate plasma and expressed that donating plasma is a good way to save a life, and it was more than for replacement plasma donors (p=0.004). The motives of most voluntary plasma donors were to contribute to society, and they believed that donating plasma is a good way, while it was not the case for most replacement plasma donors (p=0.02). Voluntary donors were more eagerly willing to donate plasma to help COVID sufferers (40.9%) when compared to replacement donors (33.2%) (p=0.037).

**Conclusion:**

Most voluntary plasma donors were regular whole blood donors and were keen to contribute to society. Convalescent plasma donation during this time of grief and loss was considered a moral responsibility by voluntary donors. The impact of media was more highly perceived in voluntary plasma donors when compared to replacement donors.

## Santrauka.

**Tikslas:**

Savanoriškos ir pakaitinės kraujo donorystės požiūrio, motyvų ir skirtumų suvokimas yra pagrindinis kelias į tvarų šio vertingo ištekliaus prieinamumą. Šiuo tyrimu buvo siekiama įvertinti nuo COVID-19 pasveikusių plazmos donorų motyvus ir požiūrį į imuninės plazmos (CP) donorystę ir suvokti savanoriškos ir pakaitinės donorystės skirtybes.

**Medžiagos ir metodai:**

Šio prospektyvaus skerspjūvio tyrimo imtis –500 nuo COVID-19 pasveikusių kraujo donorų, kurie lankėsi dėl CP donorystės tretinės sveikatos priežiūros specializuotame centre šiaurės Indijoje. Tyrimo duomenys buvo renkami naudojant internete pateiktą psichiatrijos, transfuzinės medicinos ir epidemiologijos ekspertų patvirtintą, remiantis donorų požiūriu, motyvais ir įsitikinimais, struktūruotą klausimyną.

**Rezultatai:**

Tyrimo duomenimis, savanoriai plazmos donorai anksčiau buvo reguliarūs kraujo donorai (36,8 proc.), pakaitinių plazmos donorų tokių buvo 26,4 proc. Beveik visi savanoriai plazmos donorai (99,5 proc.) nurodė altruistines priežastis, kodėl aukoja plazmą, ir teigė, kad plazmos donorystė yra geras būdas išgelbėti gyvybę, palyginti su pakaitiniais plazmos donorais (p = 0,004). Daugumos savanorių plazmos donorų motyvai buvo padėti visuomenei, jų įsitikinimu, plazmos donorystė yra geras būdas tai daryti, palyginti su pakaitiniais plazmos donorais (p = 0,02). Savanoriai donorai buvo pasirengę aukoti plazmą, kad padėtų COVID sergantiems pacientams (40,9 proc.), pakaitiniai donorai – (33,2 proc.) (p = 0,037).

**Išvada:**

Dauguma savanorių plazmos donorų buvo nuolatiniai kraujo donorai ir norėjo prisidėti prie visuomenės gerovės. Imuninės plazmos donorystė šiuo sielvarto ir praradimų laikotarpiu savanorių plazmos donorų buvo laikoma moraline atsakomybe. Žiniasklaida padarė didesnę įtaką savanoriams plazmos donorams, nei pakaitiniams donorams.

## Introduction

Convalescent plasma (CP) from the individuals who are recovered from COVID-19 has gained increasing interest in treating SARS-CoV-2 infection. [1,2,3,4] The transfusion of convalescent plasma is based on its observed utility by improving the chances of survival of patients infected with SARS-CoV-2 infection, which is under investigation. However, recent trials suggest evidence of the benefits of transfusion if CP is used early in the course of the disease [1]. It is postulated that CP acts by providing passive immunization by transferring specific neutralizing antibodies to COVID-19 patients [5]. These virus-specific neutralizing antibodies could accelerate virus clearance, limit virus entry into target cells, and prevent replicating the virus in the host [6]. The advantages of CP include ease of production, rapid deployment, specificity against the target infectious agent, immediate improvement in respiratory parameters, shortened clinical recovery time, and scalability [7]. CP has been used on a large scale globally and in India [8]. RecruitingCOVID-19 convalescent plasma donors during this pandemic has been challenging due to inexperience and differences in profile compared to whole blood donors. The scarcity of eligible recovered COVID-19 donors, recent recovery from a life-threatening disease, repeated calls and visits by health authorities and donor recruiters, fear of health, family pressure, and fear of a new procedure may add to the difficulties in converting these COVID-19 recovered patients into potential convalescent plasma donors [9].

It is important to explore various motivational factors among prospective donors for plasma donation. Blood components are a precious resource because they are obtainable only from the individuals who donate blood or its components. There are two types of donors: a voluntary nonremunerated donor and a family/replacement donor. Voluntary donors are recognized as the safest donors because they are motivated by altruism, the desire to help others, and a sense of moral and social responsibility [10]. Altruism is defined as “a selfless concern for the well-being of others”. The attitude of a blood donor is a basic evaluative judgment on a feeling or opinion about the blood donation process. It is considered that the motives of voluntary donors may differ from the motives of replacement donors.

Further, understanding this specific group of donors who have recently recovered from the potentially life-threatening disease can guide the transfusion community to enhance the base of collection of convalescent plasma. Hence the present survey was conducted to compare the motives, beliefs, and attitudes of voluntary and replacement plasma donors. The data collected during this survey will give us useful insights into the motives behind voluntary donations and how they are different from replacement donors.

## 1. Materials and Methods:

### 1.1. Study design and settings:

A prospective cross-sectional survey-based study by online medium using survey monkey platform in the COVID-19 recovered blood donors who visited for convalescent plasma (CP) donation at our tertiary care super-speciality centre and country’s first plasma bank in the national capital region of India [11]. A total of725 donors were offered to fill the study performed from 15November 2020 to 15December 2020. Donors who refused to participate or provide informed consent and were non familiar with the English language and incompletely filled donor questionnaires were excluded from the study. Finally, 456 prospective CP donors could be included in the study. The institutional ethics committee approved the study, and informed consent was taken before donor enrolment(IEC/2020/82/MA03).

### 1.2. Study objective:

This study was planned to assess the attitude and motives for convalescent plasma donation in the recovered COVID-19 plasma donors and to understand the differences between voluntary and replacement donors.

### 1.3. Inclusion and Exclusion Criteria:

**Figure 1.**
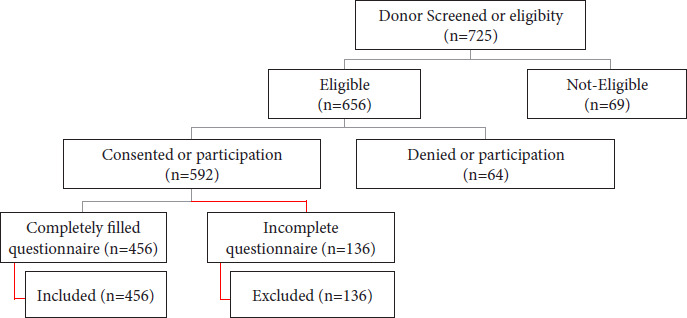
Donor participation and inclusion in the study

All the SARS-CoV-2 recovered donors who were symptomfree during the last 14 days with a negative test report for COVID-19 by RT-PCR method before donation, familiar with the English language, were included in the study survey providing additional digital consent. Donors were informed that data collected during this survey would be kept confidential and anonymous and utilized for publication in a scientific journal. Their participation was completely voluntary, and nonparticipation had no adverse consequences. Donors, who were non familiar with the English language, refused to participate or provide informed consent, and filled out incomplete questionnaires, were excluded from the study, as shown in figure 1.

### 1.4. Questionnaire:

The study questionnaire was framed by reviewing previous studies and the interactions among transfusion centers, psychiatry, and public health experts for its content validity. The demographic details of all the donors included in the study were obtained, including donor age, gender, education, occupation, and type of donation. A total of 30 questions were given to all the prospective donors to assess their motives, attitude, and belief towards convalescent plasma donation. All possible reasons were explored for determining the motives behind the donation. All questions had one of the three options based on the Likert scale offering the donors to agree, disagree and be neutral on any point of view.

### 1.5. Data collection and analysis:

The participant informed consent form was given to all the donors before providing the study questionnaire link. Data was captured and converted into a Microsoft Excel spreadsheet using “Survey Monkey” software. The original datasheet was saved as a master file in the custody of the principal investigator, and further, its duplicate versions were anonymized before sharing and analysis. Descriptive analysis was mean ± SD or median (IQR) appropriate for a continuous variable. Continuous data were analysed either by Student T-test or Mann–Whitney test depending on the normality assumption. A Chi-square test was done to assess categorical data. The 2-tailed P value less than 0.05 was considered statistically significant.

## 2. Results

The mean age of convalescent plasma donors was 32.59± 8.20 (31.99 ± 8.4 in the voluntary donors and 33.07± 7.9 in the replacement donors), and the majority were males (93.6%). The majority were married donors (58.8%) and had the education level of graduate and above (84.3%) (Table 1).

### 2.1. Voluntary and replacement donor attitude for plasma donation

The current study’s findings depicted that more voluntary plasma donors were previously regular blood donors (36.8%)when compared to replacement plasma donors (26.4%). Almost all voluntary plasma donors (99.5%) showed altruistic reasons to donate plasma and expressed that donating plasma may save a life, and it was more than for replacement plasma donors (p=0.004). Most of the voluntary plasma donors (97.4%) thought that there was nothing wrong with donating plasma, while in replacement plasma donors, few of them (7.9%) did not agree to the same (p=0.038). More voluntary plasma donors disagreed that plasma should be donated only in an emergency (51.3%), whereas most replacement plasma donors (71.5%) agreed to this or gave a neutral response (p=0.013). Statistical difference was also seen (p=0.012) between voluntary and replacement plasma donors in terms of their attitude that plasma donation is a “moral responsibility”, where 92.2% of voluntary plasma donors agreed with this attitude in comparison to 83.3% of replacement plasma donors (Table 2).

**Table 1.** Socio-demographic characteristics of Voluntary and Replacement Donors

	Sociodemographic Variables	Overall n=456 f (%)	Voluntary Donors n=193 f (%)	Replacement Donors n=263 f (%)	χ^2^	p-Value
**Age**	**Mean± SD**	32.59± 8.20	31.99 ±8.4	33.07± 7.9	1.44^#^	0.148
**Sex**	**Female**	29 (6.4)	18 (9.3)	9 (3.4)	6.931	0.008
	**Male**	427 (93.6)	175 (90.7)	254 (96.6)		
**Marital status**	**Unmarried**	186 (40.8)	92 (47.7)	95 (36.1)	8.972	0.011
	**Married**	268 (58.8)	101 (52.3)	168 (63.9)		
	**Divorced/Separated**	02 (0.4)	0	2 (0.7)		
**Education**	**Up to 12th**	72 (15.7)	28 (14.5)	41 (15.6)	7.682	0.361
	**Graduate and Above**	384 (84.3)	165 (85.5)	220 (83.7)	0.301	0.583

# independent t-test

**Table 2.** Comparison of Voluntary and Replacement Donors in terms of Attitudes regarding Plasma Donation

Sr No	Item	Voluntary Donors			Replacement Donors			p-value
		Agree	Neutral	Disagree	Agree	Neutral	Disagree	
1	I am a regular blood donor (n=454)	71 (36.8%)	38(19.7%)	84(43.5%)	69(26.4%)	82(31.4%)	110(42.1%)	**0.008**
2	I am donating plasma because I can empathize with a COVID positive person(n=455)	116(60.4%)	55(28.6%)	21(10.9 %)	159((60.5%)	73(27.8%)	31(11.8%)	0.951
3	I am donating plasma because I think it is a good way to save a life (n=456)	192(99.5%)	1(0.5%)	0	245(93.2 %)	17(6.5%)	1(0.4%)	**0.004**
4	I think there is nothing wrong with donating your plasma(n=456)	188(97.4%)	5(2.6%)	0	242(92%)	18(6.8%)	3(1.1%)	**0.038**
5	I think it was not necessary for me to donate plasma (n=455)	35(18.1%)	54(28%)	104(53.9 %)	55(21 %)	66(25.2%)	141 (53.8%)	0.675
6	I think Plasma donation is an act of altruism (n=454)	74(38.5%)	88(45.8 %)	30(15.6%)	88(33.6%)	134(51.1 %)	40(15.3%)	0.494
7	It is my religious duty to donate plasma(n=454)	73(38%)	53(27.6%)	66(34.4%)	114(43.5%)	72(27.5%)	76(29 %)	0.4
8	It is my national duty to donate plasma (n=454)	138(71.5%)	41(21.2%)	14(7.3%)	187(71.6%)	49(18.8%)	25(9.6%)	0.595
9	I feel plasma should be donated only in emergencies(n=456)	33(17.1%)	61(31.6%)	99(51.3%)	75(28.5%)	80(30.4%)	108(41.1%)	**0.013**
10	I think people who donate plasma may become infected because of needle insertion (n=456)	15(7.8%)	57(29.5%)	121(62.7%)	17(6.5%)	61(23.2%)	185(70.3%)	0.226
11	I should be paid for donating plasma (n=456)	6(3.1%)	32(16.6%)	155(80.3%)	18(6.8%)	35(13.3%)	210(79.8%)	0.152
12	Plasma donation saves life (n=456)	181 (%)	11(%)	1(%)	245(%)	17(%)	1(%)	0.924
13	I think person donating plasma should receive extra recognition in society (n=455)	48(25%)	78(40.6 %)	66(34.4%)	82(31.2%)	84(31.9%)	97(36.9%)	0.133
14	I feel plasma donation is a moral responsibility (n=456)	178(92.2 %)	14(7.3%)	1(0.5%)	219(83.3%)	36(13.7%)	8(3%)	**0.012**
15	I think the best way to donate plasma is voluntary and nonremunerated(n=455)	155(80.3%)	34(17.6 %)	4(2.1 %)	193(73.7%)	63 (24%)	6(2.3 %)	0.244

### 2.2. Motives of voluntary and replacement donors regarding plasma donation

Findings related to plasma donation motives depicted that most voluntary plasma donors wanted to “contribute to society” and thought donating plasma was a good way to make this contribution, while it was not the case for most replacement plasma donors (p=0.02). This was the only motive found statistically different between these two types of plasma donors (Table 3).

### 2.3. Believes in voluntary and replacement donors regarding plasma donation

Most of the voluntary plasma donors (96.4%) believed that plasma donation is a “good deed” as compared to the replacement plasma donors (92%), at p=0.015. Voluntary plasma donors were more willing to donate plasma to help COVID sufferers (40.9%)as compared to replacement donors (33.2%), at p=0.037(Table 4).

## 3. Discussion

The benefits of using convalescent plasma are still under investigation with its variable response in the patients to whom it is transfused. Further, the timing of plasma transfusion from days of exposure or hospitalization is under investigation in many studies. Variable treatment responses are found with different treatment modalities, and none of the treatments was effective in curtailing the mortality except dexamethasone[12]. On review of many trials, we noticed transfusion was given in the patients after three days of COVID-19 infection, which is against the principle of passive antibody transfer and might be the reason for treatment ineffectiveness. Providing passive antibodies through convalescent plasma in COVID-19was expected as one of the treatment approaches in the absence of available definitive treatment [1].

The practical challenge in convalescent plasma therapy is collecting convalescent plasma from recovered patients/donors. The challenge is to motivate, convince, and educate the potential donors and society about the likely benefits of COVID-19 convalescent plasma [13]. In India, where there is limited awareness about voluntary blood donation, motivating recovered patients to donate plasma is a big challenge [9]. One such explanation known as self-determination theory proposes that people are more likely to persist internally than externally motivated behaviors. This theory views behavior as existing on a self-determination continuum ranging from nonregulated behavior (a motivation, characterized by non-action or a complete lack of intent) to autonomous, intrinsically motivated behavior (characterized by action for the interest, enjoyment, and inherent satisfaction) [14]. In the previous study on COVID donors, we observed that COVID recovered patients who are internally motivated are more likely to come forward and help others by donating their plasma than those who are not motivated internally [15].

Our study found that around one-third of participating donors were also motivated by the COVID-19 testing, reflecting test-seeking behavior and awareness and concern about their COVID-19 antibody level to ensure their safety before plasma donation. The major reason was that only a few centres were appropriately equipped for antibody testing, so donors with the post-COVID-19 phase were keen to know their antibody levels as a marker of their immunity against this infection for future safety. Further in our centre, it was free of cost for the donors, while testing rates were high in other private laboratories.

The donors in our study were majorly replacement donors. The same pattern of donation has been seen even in blood donors. The study conducted on replacement and voluntary blood donors by Abdel et al. depicted that out of the total, 87.7% donors were replacement donors, whereas just 12.3% were voluntary donors [16]. Another study has reported that India has still a very low level of voluntary blood donors, i.e., 8 for every 1,000 of the population [17]. In a country like ours where comprehensive laboratory tests are neither possible nor pragmatic, the target is to achieve more than 90% of voluntary blood donors [18]. Nationwide lockdown, fear of getting infected, and concerns of visiting a hospital during a pandemic could have been attributed to this finding.

**Table 3.** Comparison of Voluntary and Replacement Donors in terms of Motives regarding Plasma Donation

Sr No	Item	Voluntary Donors			Replacement Donors			p-value
		Agree	Neutral	Disagree	Agree	Neutral	Disagree	
1	I want to donate plasma because it makes me feel good about myself (n=456)	172 (89.1%)	20 (10.4 %)	1 (0.5%)	225 (85.6 %)	34 (12.9 %)	4 (1.5 %)	0.406
2	I always wanted to contribute to society and donating plasma is a good way to do that(n=455)	181 (94.3%)	11 (5.7%)	0	229 (87.1%)	29(11 %)	5 (1.9 %)	**0.02**
3	I am donating plasma due to peer pressure(n=456)	15 (7.8%)	20 (10.4 %)	158 (81.9%)	33 (12.5 %)	23 (8.7 %)	207 (78.7%)	0.239
4	I am donating plasma out of my curiosity(n=452)	75 (39.3%)	64 (33.5%)	52 (27.2 %)	96 (36.8%)	76 (29.1 %)	89 (34.1%)	0.281
5	I am donating plasma because I will get a certificate(n=456)	35 (18.1%)	48 (24.9 %)	110 (57%)	43 (16.3%)	55 (20.9%)	165 (62.7%)	0.452
6	I am donating plasma because a very closely related person has also donated (n=456)	29 (15%)	40 (20.7%)	124 (64.2%)	59 (22.4%)	54 (20.5%)	150 (57%)	0.127
7	I can undergo a free Covid test due to plasma donation (n=454)	59 (30.7%)	37 (19.3 %)	96 (50 %)	92 (35.1%)	45 (17.2%)	125 (47.7%)	0.598
8	I may have some positive effect of donation on my health (n=454)	55 (28.5 %)	86 (44.6%)	52 (26.9%)	72 (27.6%)	103 (39.5 %)	86 (33%)	0.36
9	I am donating plasma because our government has insisted(n=453)	45 (23.4%)	51 (26.6%)	96 (50 %)	42 (16.1%)	75 (28.7%)	144 (55.2%)	0.146
10	I want to help someone by donating plasma (n=456)	189 (97.9 %)	3 (1.6%)	1 (0.5%)	250 (95.1 %)	10 (3.8 %)	3 (1.1 %)	0.278

**Table 4.** Comparison of Voluntary and Replacement Donors in Terms of Believes regarding Plasma Donation

Sr No	Item	Voluntary Donors			Replacement Donors			p-value
		Agree	Neutral	Disagree	Agree	Neutral	Disagree	
1.	I feel plasma donation is a good deed (n=456)	186 (96.4%)	4 (2.1%)	3 (1.6%)	242 (92 %)	20 (7.6 %)	1 (0.4%)	**0.015**
2.	I was encouraged by the number of people who are donating plasma(n=445)	91 (47.4%)	49 (25.5 %)	52 (27.1 %)	122 (48.2 %)	72 (28.5%)	59 (23.3 %)	0.612
3.	I got encouraged by the media to donate plasma to help COVID sufferers(n=455)	79 (40.9%)	44 (22.8%)	70 (36.3%)	87 (33.2%)	88 (33.6%)	87 (33.2 %)	**0.037**
4.	I am coming to donate because I was getting so many calls from the Hospitals and Govt. (n=456)	12 (6.2%)	29 (15%)	152 (78.8%)	10 (3.8%)	24(9.1 %)	229 (87.1 %)	0.061
5.	I believe people who donate plasma are temporarily weakened (n=456)	13 (6.7%)	60 (31.1 %)	120 (62.2 %)	22 (8.4 %)	77 (29.3 %)	164 (62.4 %)	0.776

The study observed that most voluntary plasma donors were regular blood donors and wanted to contribute to society. They thought that donating plasma was a good way for this contribution, while it was not the case for most replacement plasma donors. Almost all voluntary plasma donors showed altruistic reasons to donate plasma, expressing that donating plasma is a good way to save a life. It was also considered that donating plasma is a moral responsibility by voluntary donors. These findings are in line with various surveys conducted across the globe in which blood donation by donors is regarded as either moral responsibility or altruism [19]. In the study by Mishra et al., it was found that the majority (371,74.2%) of students mentioned moral responsibility as a reason for their blood donation, followed by altruism [20].

Most donors in our study were altruistic and might be more likely to donate, subjecting the study to sampling bias. Further, all plasma donors could not be assessed for their attitudes and motives regarding COVID-19 plasma donation. The participation of donors was kept voluntary and was based on getting fair participation in the study, which was explained to them when filling out the questionnaire. On analysis, we found that few donors who started filling the study questionnaire didn’t fill it completely, which might be due to their turn for donation, or they were reluctant to fill in formalities. Still, we could get enough participation from plasma donors who were representative of the plasma donor population of northern India. Also, the study involved only participants who could read and understand the English language, further limiting the enrolments. The current study was carried out in a single centre, limiting the generalizability of findings. Additionally, the study was conducted during a period when the role of convalescent plasma therapy was still evolving, which might have influenced the actual perception.

Overall, the authors found only limited numerical differences in motivating factors between donor groups. In the scenario of COVID-19 peak when it was quite clear that people may get repeated infection with COVID-19, the majority of the motivating factors didn’t work. Most voluntary donors of altruistic behavior came forward for humanity as CP therapy in the Indian subcontinent was seen as little hope to save other’s life during the COVID-19 pandemic, while replacement donors admitted they were donating CP for their patient needs.

Interestingly, our study showed that voluntary plasma donors were more encouraged by the media to donate plasma to help COVID sufferers than replacement donors. This belief of donors emphasizes the positive role of media during a pandemic. The positive part of social media in transfusion services has been detected in earlier studies. Social media has been considered a valuable tool to recruit the young donor population for whole blood donation [21,22]. This encouraging finding on the role of media may be used by transfusion services, healthcare settings, or the government to enhance the recruitment of plasma donors. Hence future studies, especially in light of changing shreds of evidence for the use of convalescent plasma therapy, are strongly recommended.

## 4. Conclusion

The majority of the voluntary plasma donors were regular whole blood donors and were keen to contribute to society. Convalescent plasma donation during this time of grief and loss was considered a moral responsibility by voluntary donors. The media impact was highly perceived in voluntary plasma donors compared to replacement donors during adversities.
